# An Italian Quack Doctor at Home

**Published:** 1887-04-02

**Authors:** 


### An Italian Quack Doctor at Home.

By an Eye-witness.

During my recent stay in the Riviera the news came that there was a fair in the little town of Rapallo, and we all went to see. The spectacle was highly amusing and interesting, but the crown and perfection of all was the Doctor. We passed through the town, as unlike as possible to an English one of the same size. There are hardly any small poor houses, all are built in four or five stories, and adorned with utter disregard of Ruskin's precepts concerning honesty and reality. One has sham pillars of imposing height with Corinthian capitals and garlands hanging between, mounted on an arcade, the whole painted in dark brown on yellow ochre walls and with large patches near the ground, where the rough grey material stands confessed. Another has nine statues on brackets, of Raffaelle, Volta, and other Italian worthies, all painted with mock shadows, in the same colours, red, brown, and yellow ochre being probably cheapest. Some have bosses and sham balconies, others have pictures commemorating the local legend of the Virgin appearing to a working man and commanding him to erect a monastery on Monte Allegro, a height where no one would build or reside, except under the sternest compulsion. A very favourite picture displays an abbess lying dead, the Trinity in the chamber, and roses strewing the floor. All these adornments of divers colours look lively and cheerful, and so do the children's garments, red, white, and blue, well washed and hung to dry from most of the upper windows, like tapestry at a royal entrance. There were booths in the streets, with cakes, and sweets, and toys; others with dress materials, often of English make, and spectacles and trinkets, while pedlars were vaunting incomparable pocket-handkerchiefs at two-pence apiece. I was familiar with the little town in its ordinary aspect, but this was a special occasion, and a number of shepherds had come down from their mountain solitudes, staring around like savages, and adorned with the hugest noses ever seen on human faces. Animals were there too, in plenty, brought for sale and barter; pretty dove-coloured cows, so small that I noticed the head of one only reaching up to the jacket of a man standing by. They were not so meek as they looked, but had plenty of frisk in them. So had the pigs. There was a lad kneeling and holding two of them fast by the hind legs, and talking with an old woman, the calm on his classic features quite unruffled by their frantic squeals and struggles. But by far the thickest crowd was assembled round a large carriage and four, from which the horses had been taken out. The vehicle was arranged as a platform, and stationed close to the sea.

There were steps on each side, a table in front with a polished metal ewer and glasses, and a stock of cases containing medicine and salve. At the back was a seat for patients, and behind that were stationed four musicians with wind instruments. In the midst stood the portly figure of *Professor Gandolfi*, a tall, stout man of middle age, respectably bald, his face clean shaven except the thick moustache, and wearing the whitest of linen, and the blackest of broadcloth. He spoke pure Italian, without a shade of dialect, articulated clearly, with no bawling or hesitation, and gesticulated so freely and gracefully with his right hand that one did not at first observe that the other sleeve was empty. When we drew near to listen, he was expatiating on his own surpassing skill as a dentist, and inviting all the people to come up by this stair, and go down by that stair—he would draw their teeth without any charge—in an instant, and they should go home without any teeth at all. The crowd laughed, and, allured by so pleasing a prospect, a rough, silly-looking shepherd lad came up, seated himself, and opened his mouth with such goodwill that the by-standers could see down his throat. The Professor must have been really skilful, for after a little fumbling with his one hand, out came the tooth, which he presented to its owner with a bow, and the poor lad with his hand to his jaw stumbled down, followed to the edge by the urbane Professor with a lesson of politeness: "Thanks, dear sir, many thanks. O no, you need not thank me, happy to be of use." And to the delight of the crowd this lesson was repeated every time an unlucky sufferer forgot his manners.

Other patients followed in a pretty continuous stream, old and young, men and women, and as continuous was the flow of the Professor's eloquence. Sometimes the patient sat waiting five or ten minutes, and always with perfect ease and self-possession. What would induce an English rustic to go through such an ordeal? A little girl of about eleven came up, and with her the great man was very gentle, while the musicians played a soft and soothing strain of consolation. To the young men he discoursed concerning valour, and the glory of their ancestors, and the trumpets blew inspiriting marches. I wondered at his extracting all these teeth gratis, but presently perceived how his profit was to come; for as the patients became fewer, he said that drawing teeth was to him "nothing, a mere nothing as they could see—he wished they would bring him something more difficult—a cancer, a polypus in the nose—that was what he would really like to treat." He described various kinds of catarrh, illustrating each by its appropriate cough, and explained the course and danger of many other diseases, patting and stroking those parts of his ample person which were afflicted by each malady. Of course, every ailment internal or external was to be speedily cured by his remedies, the herbs composing which had been collected in the mountains by his own peasants, well-to-do folks.

The actual business of the day was now in full swing. The doctor had stationed his coachman at one stair to receive the now indispensable fees, while he himself at the other handed down the medicine and salve. This coachman was an imposing personage in livery, with a cockade in his hat; he was tall, thin, and mute as the grave, but his visage wore a most sardonic grin under his hanging moustache. He contrasted finely with his eloquent master. In a very short time as much as £4 was taken. We came again in the afternoon, and still the flow of words was pouring out, and the money as freely pouring in, so the Professor's visit to Rapallo must have been satisfactory to himself. Towards the end he invited any one who had been hurt to present himself, and he would give him £4. No one appeared, and, indeed, no one had uttered a sound during the tooth-drawing; only one poor lad put up his hand, and the Professor gave it a smart pat, accompanied by a lecture to the victim, and an appeal to the bystanders.

When we returned in the afternoon he had a rival holding forth at a little distance—a woman; she wore a half-mask, and carried a black sunshade, so that expression and gesticulation were both wanting to her monotonous discourse. She professed to tell fortunes; but although the young people giggled and looked sheepish, we saw no one consult the sorceress, both honour and profit remaining with the incomparable *Professor Gandolfii.* His figure and its surroundings—the women with veiled and kerchiefed heads—the men with cloaks and sashes—the booths with their smart contents at a little distance—the clear Italian sky, and the sunny Mediterranean as a background—all formed a picture not to be seen elsewhere.

